# Quality of care of treatment for uncomplicated severe acute malnutrition provided by lady health workers in Pakistan

**DOI:** 10.1017/S1368980017002610

**Published:** 2017-10-27

**Authors:** Eleanor Rogers, Muhammad Ali, Shahid Fazal, Deepak Kumar, Saul Guerrero, Imtiaz Hussain, Sajid Soofi, Jose Luis Alvarez Morán

**Affiliations:** 1 Action Against Hunger, 161–163 Greenwich High Road, London SE10 0JA, UK; 2 Action Against Hunger, Islamabad, Pakistan; 3 Action Against Hunger, New York, NY, USA; 4 Department of Pediatrics and Child Health, Aga Khan University, Karachi, Pakistan

**Keywords:** Community-based management of acute malnutrition, Severe acute malnutrition, Community health workers

## Abstract

**Objective:**

To assess the quality of care provided by lady health workers (LHW) managing cases of uncomplicated severe acute malnutrition (SAM) in the community.

**Design:**

Cross-sectional quality-of-care study.

**Setting:**

The feasibility of the implementation of screening and treatment for uncomplicated SAM in the community by LHW was tested in Sindh Province, Pakistan. An observational, clinical prospective multicentre cohort study compared the LHW-delivered care with the existing outpatient health facility model.

**Subjects:**

LHW implementing treatment for uncomplicated SAM in the community.

**Results:**

Oedema was diagnosed conducted correctly for 87·5 % of children; weight and mid upper-arm circumference were measured correctly for 60·0 % and 57·4 % of children, respectively. The appetite test was conducted correctly for 42·0 % of cases. Of all cases of SAM without complications assessed during the study, 68·0 % received the correct medical and nutrition treatment. The proportion of cases that received the correct medical and nutrition treatment and key counselling messages was 4·0 %.

**Conclusions:**

This quality-of-care study supports existing evidence that LHW are able to identify uncomplicated SAM, and a majority can provide appropriate nutrition and medical treatment in the community. However, the findings also show that their ability to provide the complete package with an acceptable level of care is not assured. Additional evidence on the impact of supervision and training on the quality of SAM treatment and counselling provided by LHW to children with SAM is required. The study has also shown that, as in other sectors, it is essential that operational challenges are addressed in a timely manner and that implementers receive appropriate levels of support, if SAM is to be treated successfully in the community.

Severe acute malnutrition (SAM) is a major global public health challenge, with an estimated 16·5 million children under 5 years of age suffering from the condition^(^ ^1^ ^)^. Currently, treatment is available for only about 3 million children per year, leaving a significant treatment deficit^(^ ^2^ ^)^. Treatment is provided primarily through the Community Management of Acute Malnutrition (CMAM) model. Nurses provide treatment during weekly visits to health centres which is continued in the home using ready-to-use therapeutic food (RUTF). This outpatient model has successfully reduced the burden on carers compared with inpatient care and therefore limited the proportion of children defaulting from the programme while increasing treatment coverage^(^ ^3^ ^)^. Despite this success, treatment coverage of CMAM services remains low, often with less than 40 % of intended recipients receiving care^(^ ^4^ ^)^. The main barriers to access are a lack of knowledge about malnutrition and the availability of treatment among carers, and the high opportunity cost which continues to be associated with receiving treatment^(^ ^4^ ^–^ ^6^ ^)^.

In multiple contexts, diarrhoea, pneumonia and malaria treatment is delivered by community-based actors through the integrated Community Case Management (iCCM) and the Community-based Integration of Childhood Illnesses (C-IMCI) models^(^ ^7^ ^)^. Community health workers (CHW) are trained to screen for, classify and treat these three conditions in their own communities. Proximity of treatment to the community is the main benefit of this approach with community members more likely to access treatment if it is available in their own community, reducing the financial and temporal barriers associated with seeking treatment in a health centre^(^ ^8^ ^,^ ^9^ ^)^. This reduces the severity of disease as cases are identified and treated earlier^(^ ^10^ ^)^. Moreover, evidence shows that CHW are able to provide high-quality treatment^(^ ^11^ ^)^.

In a handful of contexts, SAM treatment has successfully been added to the standard iCCM package, with CHW proven to be able to provide care which is non-inferior to that provided at the health centre. In Bangladesh, CHW delivered an acceptable level of care with 89·1 % of CHW providing at least 90·0 % error-free case management^(^ ^12^ ^)^. In Ethiopia, treatment was delivered by health extension workers alongside malaria, measles, diarrhoea and pneumonia treatment^(^ ^13^ ^)^. This service, delivered at scale in Oromia Region, managed 59·0 % of malnutrition cases correctly. In Mali, 79·5 % of cases were correctly assessed, classified and treated by CHW^(^ ^14^ ^)^. In addition, the approach has been credited with promoting early case finding^(^ ^12^ ^)^.

To understand whether SAM treatment could be effectively delivered in the community in Pakistan, Action Against Hunger and Aga Khan University, in agreement with the Department of Health, tested the feasibility of the implementation of screening and treatment of uncomplicated SAM in the community by lady health workers (LHW). The research aimed to address the question of whether CHW can offer quality care in the community for children suffering from uncomplicated SAM, by adequately assessing their nutrition status, identifying danger signs and complying with protocols for the provision of therapeutic treatment. To assess this, SAM treatment was added to the existing basic package of LHW-delivered child health interventions. Between March 2015 and April 2016 in the south-east of Pakistan in Dadu district, Sindh Province, an observational, clinical prospective multicentre cohort study was implemented with an intervention and control arm comparing the LHW-delivered care for uncomplicated SAM with the existing outpatient health facility model (CMAM).

The current paper presents the findings of a cross-sectional study assessing the technical competence – quality of care – of LHW managing cases of uncomplicated SAM in the community. The study evaluated the capacity of LHW to determine the nutritional status of children aged 6–59 months; to identify danger signs in a malnourished child and refer them if necessary; to treat cases of SAM without complications according to protocol; and to provide nutritional counselling.

## Materials and methods

The quality-of-care study reported herein was part of a two-arm cluster-randomized trial which used restricted stratified randomization to allocate clusters to the two arms. A total of six Union Councils were covered; three as the control arm and three as the intervention arm. In the intervention area, seventy-two LHW screened for both complicated and uncomplicated SAM, treated cases of uncomplicated SAM in their village and referred any complicated cases to the health centre. LHW provided treatment in the community ‘health house’ which is the house of the LHW and located in each village, approximately a 10 to 25 min walk from each household. All LHW were female and belonged to an existing government programme under which each LHW was attached to a government health facility, from which they received a government allowance (R15 000 or $US 142/month) and medical supplies to support 1000 people within a catchment area of 200 households. All LHW have a minimum of 8th grade standard formal education, and received 2 years of training on family planning and basic child health when joining the scheme. Under this government programme, the LHW provide information to the community on family planning and screening and referrals to the health centre for basic health services. Additionally, they participate in any community health campaigns as required but do not perform any SAM screening or referral activities under the existing government scheme.

For the current intervention, LHW were trained by Action Against Hunger on CMAM protocols, SAM case management, and infant and young child feeding. After an interval of 3 to 6 months, a refresher on the updated CMAM guidelines was provided. LHW supervisors monitored the LHW once per month and three Action Against Hunger nurses supervised them twice weekly. Action Against Hunger also ensured provision of the essential supplies at health facilities and health houses. LHW did not receive an additional salary for delivering additional services as part of this trial. Pre-existing, facility-based services remained open in the intervention arm throughout the pilot period. In the control arm, LHW continued to carry out their tasks under the government scheme, as previously outlined. The cohort study examined the impact of integrating the screening and treatment of SAM into the LHW’s existing role. Specifically, the intervention’s effectiveness (i.e. cure, default and death rates) and coverage (i.e. proportion of eligible cases receiving treatment), the quality of care provided by the LHW and the cost-effectiveness of the intervention were assessed. The trial was conducted between April 2015 and July 2016.

The cross-sectional quality-of-care study presented in the current paper was conducted between May and June 2016. The study was designed using existing WHO guidance^(^ ^15^ ^)^ and previous quality-of-care studies on CHW^(^ ^13^ ^)^. The study focused on the care provided by a sub-set of seventeen LHW across the three Union Councils of the pilot’s intervention arm. As the study was undertaken towards the end of the implementation period, only eighteen CHW were still active and one of these was unavailable for the full duration of the study.

Quality of care is defined as the capacity of the CHW to evaluate, classify and treat cases of uncomplicated SAM, to provide nutritional counselling to carers of children receiving treatment for SAM, and to refer complicated cases of SAM to inpatient care. In Pakistan, in children under 5 years of age, uncomplicated SAM is defined as mid upper-arm circumference (MUAC) <11·5 cm and/or nutritional oedema. Children with appetite and no medical complications are provided with weekly rations of RUTF and are assessed weekly during follow-up. Children were discharged from treatment when their MUAC was 125 mm. In Dadu, as there is no treatment available for moderate acute malnutrition, discharged cases were provided with one sachet per day in the follow-up period which lasted for 2 weeks, to minimize relapses.

Quantitative data were collected through an observational survey with independent enumerators using a checklist to observe LHW work. The grading of LHW competence involved the observers watching each LHW perform specific tasks and identifying whether they were done correctly. Each task was broken down by each required step as defined by the treatment protocol. If the LHW completed all steps in the process correctly, she was deemed able to correctly conduct that task. For the MUAC measurement, the observers also took their own measurement and compared it with the LHW’s. If the LHW’s measurement was within a 2 mm margin of the observer’s measurement, it was deemed correct. This was complemented by a review of admission cards and registers to assess completeness and accuracy of health records. Five teams of two enumerators observed LHW care during the 2-week data collection period. Enumerators had a nursing diploma and had worked in CMAM for a minimum of 4 years. They were trained on the study assessment technique for 3 d, with role plays and discussions around ‘good’ *v*. ‘poor’ practice for each checklist item. The training included two standardization tests to ensure a concordance with the gold standard of 90 % or more by each enumerator. Informed consent was obtained from all LHW and carers who participated in the study.

Data were entered into EpiData software version 3.1 and analysed with the R Language for Statistical Computing version 3.2.5. A composite indicator was built combining essential indicators from the treatment component. This composite indicator can be considered the minimum set of tasks that are essential for the LHW to treat an uncomplicated case of SAM and includes multiple components of the medical and nutrition treatment.

## Results

The quality of care provided by a total of seventeen LHW was assessed. They had a median age of 36 years, ranging from 21 to 45 years. All had a minimum of 8 years of school education, essential criteria for all LHW working in Pakistan.

The treatment of sixty-one SAM cases was observed during the study. Table 1 outlines the characteristics of the cases assessed.

**Table 1:** Characteristics of cases observed in the quality-of-care study in the intervention area, Dadu district, Sindh Province, south-east Pakistan, May–June 2016

Characteristics of cases observed		*n*	%
Age	06–23 months	37	60·7
	24–59 months	24	39·3
Sex	Male	26	42·6
	Female	35	57·4
Type of case	New cases	12	19·7
	Cases already in the programme	49	80·3
	Total	61	100·0

Of the sixty-one cases, twelve were new to treatment and forty-nine were already receiving treatment for SAM at the time of assessment. The median age of all cases observed was 21 months, with a range of 7 to 58 months; 60·7 % of cases were less than 2 years old; and nearly 60·0 % of cases assessed were female.

The results of the assessment of cases by the LHW are presented in Table 2.

**Table 2:** Selected indicators of quality of case management by LHW in the quality-of-care study in the intervention area, Dadu district, Sindh Province, south-east Pakistan, May–June 2016

	Number tested	*n*	%
General assessment			
Child correctly evaluated for the main danger signs	60	39	65·0
Child’s respiratory rate counted*	50	19	38·0
Child’s temperature checked	60	39	65·0
Child assessed correctly for dehydration	58	28	48·3
Nutrition assessment (all cases, new and follow-up)			
Child with oedema are correctly evaluated*	16	14	87·5
Child’s MUAC is correctly measured†	61	35	57·4
Child’s weight is correctly measured	60	36	60·0
Appetite test is correctly performed	50	21	42·0
Nutrition assessment (new cases only, *n* 12)			
Child with oedema are correctly evaluated*	6	6	100·0
Child’s MUAC is correctly measured†	12	10	83·3
Child’s weight is correctly measured	12	7	58·3
Nutrition treatment (new cases only, *n* 12)			
Antibiotics given according to protocol	12	8	66·7
Folic acid given according to protocol	5	4	80·0
Nutrition treatment (all cases)			
RUTF given according to protocol‡	50	36	72·0
Carer receives all counselling messages correctly	61	4	6·6
Child receives correct nutrition and medical treatment§	50	34	68·0
Child receives correct nutrition and medical treatment plus carer receives key counselling messages correctly	50	2	4·0

LHW, lady health workers; MUAC, mid upper-arm circumference; RUTF, ready-to-use therapeutic food.

* LHW were informed that if the child looks well, they were not required to check this.

† ≤2 mm margin of error between LHW and observer measurements.

‡ The weighing machine to measure RUTF was not functioning for all LHW, so some could not provide RUTF and therefore could not be observed doing so during the study.

§ Folic acid, antibiotics and RUTF provided according to the protocol.

Danger signs were correctly assessed for 65·0 % of all cases, as was the child’s temperature. The respiratory rate and dehydration checks were carried out correctly for 38·0 % and 48·3 %, respectively.

There was variation in the proportion of cases that were correctly assessed for the four components of the nutrition assessment. Oedema was checked correctly for the highest proportion of cases (87·5 %). However, the child’s weight and MUAC were conducted correctly for only 60·0 % and 57·4 % of cases, respectively. The appetite test was conducted correctly for 42·0 % of cases.

Of the twelve new cases assessed during the present study, 66·7 % were given antibiotics according to the protocol. Only five cases were given folic acid, due to a stock outage in the health post, with four of the five cases (80·0 %) having it administered according to protocol. Similarly, there was a stock outage of RUTF but of the fifty cases it was provided to, 72·0 % were given the correct dose and advice. All children and carers received counselling messages, but only 6·6 % (*n* 4) received them all correctly.

Of all cases of SAM without complications assessed during the present study, 68·0 % received the required medical and nutrition treatment (antibiotics, folic acid, RUTF) according to protocol. The proportion of cases that received the correct medical and nutrition treatment and key counselling messages was 4·0 %.

## Discussion

The present quality-of-care study shows that LHW in Pakistan are able to assess nutritional status and provide appropriate treatment for cases of uncomplicated SAM. LHW are able to conduct the nutrition assessment accurately, measure oedema, MUAC, weight and conduct the appetite test correctly. The majority of LHW were able to correctly assess oedema (87·5 %), which is a high proportion compared with other studies^(^ ^16^ ^)^. LHW were less competent at weight and MUAC assessment, with 60·0 % and 57·4 % of cases measured correctly, respectively. Nevertheless, of all 425 cases of SAM without complications treated by LHW in the intervention arm, 76·0 % were discharged as cured, 3·8 % were discharged as defaulters and only one death was reported^(^ ^17^ ^)^. These outcome indicators meet the international Sphere standards^(^ ^18^ ^)^, showing that LHW are able to provide care in line with that provided by the existing outpatient treatment model^(^ ^3^ ^)^.

The appetite test, traditionally performed by trained medical professionals in outpatient centres, was performed correctly in 42·0 % of cases. This supports existing evidence that minimally trained community-based workers are able to undertake this task, although as it was undertaken inaccurately for more than half of the cases, this suggests that steps need to be taken to improve consistency^(^ ^12^ ^–^ ^14^ ^)^. The composite indicator demonstrated 68·0 % of cases received medical and nutrition treatment correctly. However, when the provision of key nutritional counselling messages to the carer was included in the composite indicator, only 4·0 % of cases assessed in the present study received all three elements correctly. This shows that LHW were not accurately providing key counselling messages to the majority of cases, which programme staff reported was due to their perceived high workload, but could equally be due to insufficient training on this component. Further investigation would be required to understand the specific cause of their poor performance in this area.

A high quality of care was not consistently provided by all LHW, demonstrating that the transfer of treatment to community-based staff does not guarantee an acceptable level of quality of care and additional measures may be required to achieve it. Comparisons with previous studies suggest that the context and implementation model may have played a part. The results from the present study are lower than those reported in Bangladesh, where 89·1 % of CHW provided at least 90·0 % error-free case management^(^ ^12^ ^)^, as well as those reported from Mali where 79·5 % of cases were correctly managed^(^ ^14^ ^)^. However, they are in line with results from the government-run health extension programme in Ethiopia where 59·0 % of children with malnutrition were treated correctly^(^ ^13^ ^)^. The key difference between the Bangladesh and Mali interventions and the Ethiopia and Pakistan interventions is the operational conditions under which they were implemented. In the former, the interventions were conducted by a non-governmental organization with paid CHW and close and constant expert supervision. Yet in Ethiopia, the intervention was part of a government-run scheme with limited resources under routine operational conditions. This second scenario more closely reflects the challenges faced in the present study in Pakistan, where the human and financial resources were shared between the government and a non-governmental organization, leading to operational challenges which resulted in stock outages of key commodities such as folic acid, antibiotics and weighing machines.

Another factor which likely affected the quality of care was the reported poor motivation of the LHW. Motivation has been previously identified as a common and significant determinant of the quality of care provided by CHW^(^ ^19^ ^)^. In Pakistan, LHW accurately measured new cases significantly better than follow-up cases, with 83·3 % of MUAC measurements in new cases measured correctly compared with 57·4 % in all cases. Anecdotal evidence from programme staff and LHW supervisors suggests that motivation to treat cases when first enrolled leads to better accuracy in measuring cases, but this motivation waned over the course of the child’s treatment. This variation over time is also believed to have been influenced by the LHW belief that they were not paid sufficiently for providing this service. In Pakistan, financial remuneration is customarily paid to LHW for new activities and campaigns, but the LHW in the present study were not paid any additional sum on top of their existing salary. Therefore, it is likely and unsurprising that their motivation, and thus the quality of care they provided, decreased over the course of the child’s treatment.

The present study had several limitations. First, it did not include a qualitative component, so it is not a holistic study, providing no evidence as to whether the quality of care was acceptable for the beneficiary community or to LHW providing this additional package. Second, the sample size of the study was small, with only seventeen out of the seventy-two LHW included in the quality-of-care study, limiting the wide applicability of results. Third, close observation of LHW may have led to observer bias, as LHW are likely to have performed better than when they are conducting activities without an observer. Fourth, the quality of care provided in the control arm was not assessed, preventing a more comprehensive comparative analysis. Despite these limitations, the study findings suggest that LHW have the capacity to accurately assess nutritional status and provide treatment for uncomplicated SAM, and that additional investigation into motivation and other determinants of performance is needed before the approach can be successfully implemented at scale.

## Conclusions

The present quality-of-care study supports existing evidence that LHW are able to identify uncomplicated SAM and a majority can provide appropriate nutrition and medical treatment in the community. However, the findings also show that their ability to provide the complete package with an acceptable level of care is not assured. Additional evidence on the impact of supervision and training on the quality of SAM treatment and counselling provided by LHW to children with SAM is required. The study has also shown that, as in other sectors, it is essential that operational challenges are addressed in a timely manner and that implementers receive appropriate levels of support, if SAM is to be treated successfully in the community.

## References

[1] UNICEF, World Health Organization & The World Bank Group (2016) Joint Child Malnutrition Estimates – Levels and Trends (2016 edition). http://www.who.int/nutgrowthdb/estimates2015/en/ (accessed February 2017).

[2] UNICEF (2015) Annual Results Report: Nutrition. https://www.unicef.org/publicpartnerships/files/2015ARR_Nutrition.pdf (accessed March 2015).

[3] Collins S, Dent N, Binns P et al. (2006) Management of severe acute malnutrition in children. Lancet 368, 1992–2000. 17141707 10.1016/S0140-6736(06)69443-9

[4] Rogers E, Myatt M, Woodhead S et al. (2015) Coverage of community-based management of severe acute malnutrition programmes in twenty-one countries, 2012–2013. PLoS One 10, e0128666. 26042827 10.1371/journal.pone.0128666 PMC4456359

[5] Puett C & Guerrero S (2015) Barriers to access for severe acute malnutrition treatment services in Pakistan and Ethiopia: a comparative qualitative analysis. Public Health Nutr 18, 1873–1882. 26017477 10.1017/S1368980014002444 PMC10271649

[6] Bliss JR, Njenga M, Stoltzfus RJ et al. (2016) Stigma as a barrier to treatment for child acute malnutrition in Marsabit County, Kenya. Matern Child Nutr 12, 125–138. 25989353 10.1111/mcn.12198 PMC6860141

[7] World Health Organization & UNICEF (2012) WHO/UNICEF Joint Statement: Integrated Community Case Management (iCCM). Geneva/New York: WHO/UNICEF.

[8] Jacobs B, Ir P, Bigdeli M et al. (2012) Addressing access barriers to health services: an analytical framework for selecting appropriate interventions in low-income Asian countries. Health Policy Plan 27, 288–300. 21565939 10.1093/heapol/czr038

[9] George A, Menotti EP, Rivera D et al. (2009) Community case management of childhood illness in Nicaragua: transforming health systems in underserved rural areas. J Health Care Poor Underserved 20, 4 Suppl., 99–115. 20168036 10.1353/hpu.0.0205

[10] Kalyango JN, Alfven T, Peterson S et al. (2013) Integrated community case management of malaria and pneumonia increases prompt and appropriate treatment for pneumonia symptoms in children under five years in Eastern Uganda. Malar J 12, 340. 24053172 10.1186/1475-2875-12-340 PMC3848942

[11] Bosch-Capblanch X & Marceau C (2014) Training, supervision and quality of care in selected integrated community case management (iCCM) programmes: a scoping review of programmatic evidence. J Glob Health 4, 020403. 25520793 10.7189/jogh.04.020403 PMC4267084

[12] Puett C, Coates J, Alderman H et al. (2013) Quality of care for severe acute malnutrition delivered by community health workers in southern Bangladesh. Matern Child Nutr 9, 130–142. 22515318 10.1111/j.1740-8709.2012.00409.x PMC6860607

[13] Miller NP, Amouzou A, Tafesse M et al. (2014) Integrated community case management of childhood illness in Ethiopia: implementation strength and quality of care. Am J Trop Med Hyg 91, 424–434. 24799369 10.4269/ajtmh.13-0751 PMC4125273

[14] Alvarez Morán JL, Alé FB, Rogers E et al. (2017) Quality of care for treatment of uncomplicated severe acute malnutrition delivered by community health workers in a rural area of Mali. *Matern Child Nutr* (Epublication ahead of print version). 10.1111/mcn.12449 PMC6866144 28378463

[15] World Health Organization (2003) Facility Survey: Tool to Evaluate the Quality of Care Delivered to Sick Children Attending Outpatient Facilities. Geneva: Department of Child and Adolescent Health and Development, WHO.

[16] Alvarez JL, Dent N, Browne L et al. (2016) Putting Child Kwashiorkor on the Map. http://www.ennonline.net/childkwashiorkor (accessed October 2017).

[17] Hussain I, Channar S, Hussain A et al. (2017) Evaluation of the Effectiveness and Impact of Community Case Management of Severe Acute Malnutrition Through Lady Health Workers as Compared to a Facility Based Program: Cluster Randomized Controlled Trial. Karachi: Aga Khan University.

[18] Sphere Project (2011) The Sphere Handbook, 3rd ed. http://www.sphereproject.org (accessed March 2017).

[19] Strachan DL, Källander K, ten Asbroek AHA et al. (2012) Interventions to improve motivation and retention of community health workers delivering Integrated Community Case Management (iCCM): stakeholder perceptions and priorities. Am J Trop Med Hyg 87, 5 Suppl., 111–119. 23136286 10.4269/ajtmh.2012.12-0030 PMC3748511

